# Meeting Report: Translational Considerations of Novel Vector Management Approaches

**DOI:** 10.1371/journal.pntd.0004800

**Published:** 2016-08-11

**Authors:** Adriana Costero-Saint Denis, Wolfgang W. Leitner, Tonu Wali, Stephanie James

**Affiliations:** 1 Division of Microbiology and Infectious Diseases, National Institute of Allergy and Infectious Diseases, National Institutes of Health, Bethesda, Maryland, United States of America; 2 Division of Allergy, Immunology, and Transplantation, National Institute of Allergy and Infectious Diseases, National Institutes of Health, Bethesda, Maryland, United States of America; 3 Foundation for the National Institutes of Health, Bethesda, Maryland, United States of America; National Institute of Allergy and Infectious Diseases, UNITED STATES

## Report: Sept. 14, 2015 NIAID/FNIH joint workshop on “Translational Considerations of Novel Vector Management Approaches”

There are numerous vector control strategies under development that could be considered “novel.” These include those proposing the use of symbionts, biological agents, and new ways to use existing control tools as well as those aimed at developing new active ingredients for familiar applications such as insecticides and odorants/repellents. In some cases, the national and international development pathways are in place; for example, a standardized path for assessment of new vector control products has recently been published [1]. However, some proposed vector control methods are being designed purposely to incorporate unusual characteristics intended to facilitate deployment, sustainability, and cost-effectiveness. Persisting biocontrol methods, genetically modified vectors, and paratransgenic approaches provide examples of products that require new thinking about the testing pathway. This includes recognition that for products intended to be persistent or self-sustaining, conduct of open field trials essentially constitutes the first phase of deployment, and there will be a need for safety monitoring that extends beyond the trial [2].

In order to examine these new strategies and facilitate discussion amongst scientific experts, the National Institute of Allergy and Infectious Diseases (NIAID) and the Foundation for the NIH (FNIH) organized a workshop called “Translational Considerations of Novel Vector Management Approaches.” The goal of this workshop was to discuss novel types of vector control technology that may face difficulty fitting into an existing development pathway. This report provides a summary of the discussions that occurred at the workshop.

## Planning Considerations

Translation of conceptually novel approaches to field application can be expected to be both labor- and resource-intensive. Traditional research funding mechanisms and practices are often limited in their ability to provide adequate support for the crucial auxiliary activities discussed below. This may create a need for broader funding, and perhaps operational, consortia. Before commencing this type of research, careful thought should be given to whether the potential utility of the anticipated product justifies the necessary investment in its development. Is there a widely recognized public health need that the novel product might be uniquely poised to meet? Are there conditions under which the novel strategy has a competitive advantage over other currently used disease control strategies? A target product profile (TPP) is a development tool that can help guide these research decisions. The TPP focuses on the attributes that the product must have to be successful. These include characteristics such as intended use, target population, minimum effect size for public health relevance, specificity, stability, safety, ease of manufacture, and delivery. The TPP can be an important resource for both funders and regulators.

## Trial Site Requirements

Researchers seeking to develop a novel vector control approach need to consider well in advance the testing and regulatory requirements they will confront. Site selection requirements will extend beyond the basic entomologic and epidemiologic needs [3]. Early in the planning, it will be necessary to consider whether there are any social, legal, or political barriers to the new technology at the potential field site that might cause testing to be problematic and/or controversial. This will require identification of all the relevant local laws and concerned government agencies and early interactions to determine if they are open to the technology. Likewise, it is essential to be aware in advance of any significant public or institutional opposition to the research, to understand the rationale for that opposition, and to determine whether it can be addressed through the experimental design and communications plan. If there is widespread opposition to the technology that cannot be addressed adequately, further development at that site may not be feasible. Obtaining this understanding takes significant outreach efforts, for which knowledgeable local collaborators with good political and community relations will be a great asset.

Determining institutional readiness is also an important component of site selection. If contained studies are envisioned, facilities should provide for a containment level commensurate with both national regulatory and internationally accepted standards (for example, see [2,4,5]). If the proposed site is otherwise appropriate, it may still require support to improve existing facilities or even construct new ones. In gearing up for trial conduct, additional training of personnel may be necessary. If ecologically confined studies are planned, then field sites with appropriate geographic isolation must be available.

## Regulatory Considerations

It is critical that there be an established and credible regulatory system in place. An essential site selection criterion is the presence of policies and structures for biosafety, regulatory, and ethical oversight [6]. The regulatory path is usually guided by precedent, based on similar products and their intended uses. Thus, it will be helpful if the regulatory system has had experience with, for example, conventional biocontrol agents or genetically modified (GM) crops. However, where a truly novel product, such as a genetically modified microbiome, is proposed, there may be little or no precedent. Self-perpetuating methods, such as those incorporating gene drive, introduce complexities associated with potential for transboundary movement and control that may require regional solutions. These kinds of products will be new to all regulatory systems. Regulatory capabilities of disease-endemic countries can be particularly strained by such novel products, necessitating a mechanism for training and capacity strengthening of the biosafety and regulatory infrastructure while, at the same time, avoiding a conflict-of-interest situation.

## Risk Assessment and Management

Novel vector control products will be associated with uncertainties about risks to human health and the environment. This requires risk assessment that considers the potential for adverse effects from a biological and ecological perspective but also takes into account stakeholder concerns such as social and economic risks. It is recommended that projects proposing field testing of novel vector control products obtain objective independent opinions on the risks posed, including representation from those living at the testing site. Results from such a risk assessment can identify additional research that should be done or additional risk management needs before the project seeks regulatory approval for field testing. The European Food Safety Authority has published guidance on environmental risk assessment of genetically modified animals, including insects, [7] and The Convention on Biological Diversity has published guidance on risk assessment of living modified organisms [8], both of which provide insights into the kinds of scientific concerns that may be raised with respect to novel vector control technologies. These include issues of: persistence and invasiveness; horizontal gene transfer; potential influence on pathogens, infections, and disease; interactions with target and non-target organisms; management system effects; and human or animal health effects. Examples of risk assessments for release of novel mosquito products are publicly available [9,10].

The National Environmental Policy Act (NEPA) requires all United States of America federal agencies to evaluate the environmental impacts of their actions. NEPA applies to all federally funded research and development projects, including those funded by NIH, whether the research takes place in the US or other countries. At the NIH, if the funding institute or center believes a grant meets the extraordinary circumstances criteria [11], then it will be forwarded to the NIH Office of Research Facilities, Division of Environmental Protection, which will review the project and determine what level of NEPA documentation (Categorical Exclusion, Environmental Assessment, or Environmental Impact Statement) is required.

Effective risk management provides ways to avoid or reduce risk to acceptable levels based on agreed performance standards and procedures. Standard operating procedures must be developed for all aspects of a trial, and staff should be trained in compliance according to relevant standards of good practice. Stopping criteria based on both efficacy and safety requirements should be identified before each new phase of testing. Documentation and mitigation processes should be in place to provide operational assurance and responsive management. Risk characterization will be considered by national regulators, who may approve the trial or not, as well as local communities and other stakeholders, who may accept the trial or not. Specific monitoring requirements may be imposed.

## Social and Ethical Considerations

Acceptability of the research, and of the ultimate product, is paramount to its success. Examples abound where a lack of understanding of ethical, social, and cultural issues has delayed or even prevented adoption of a new product. Furthermore, a false step that arouses public concerns about a new type of technology can have ramifications far beyond the project initially involved, slowing the progress of the field as a whole. It will be important to engage early with local/regional experts who are familiar with the culture, including social scientists, to plan the stakeholder engagement strategy. No roadmap exists for effective stakeholder engagement, but it includes both an ethical component and a communications component. Local co-ownership of the entire product development and testing process, with appropriate training and technology transfer, can be key to acceptability and successful deployment of new technologies.

Translational vector biology research raises a number of challenges for the current “clinical bedside” paradigm of research ethics. Vector interventions generally are area-wide, not at the level of the individual, and involve multiple forms of interaction with stakeholders at various levels. The interests of the different types of stakeholders will vary in their ethical significance. Public safety is a cardinal public interest that is best addressed through the regulatory mechanisms imposed and overseen by government. Informed consent is required for stakeholders who meet the criteria of research subjects, such as those who provide clinical specimens or identifiable information at the personal or household level. Simply living in the vicinity of a vector release does not qualify someone as a research subject. Yet researchers are obligated to respect the interests of those who, while not research subjects, are associated with and/or affected by the trial in a meaningful way. Community engagement provides a mechanism to acknowledge and respond to this larger group of stakeholders. The precise nature of these obligations will vary from context to context, but a common principle is that stakeholders should be provided with sufficient opportunity to interact with the project team to learn about the research and its implications and to formulate reasoned positions about acceptance [2,12]. Public perception is not solely a function of information versus misinformation or lack of information, although researchers will be wise to ensure that they are making accurate information about their work widely available on an ongoing basis, or of the public’s ability to understand this information (scientific literacy). It will also have to do with how people interpret information based on their personal values and cultural identities [13]. Risk attitude, which takes into account perception of both risks and benefits of the technology, is the ultimate driver of acceptance. Framing communications about novel technologies in ways that do not play into divergent worldviews offers the best chance of meaningful discussion.

It must be emphasized that adequate engagement goes beyond just informing stakeholders about the project. Engagement must involve bi-directional exchanges, which commit those developing the technology to listening and learning from the community. Researchers must be willing to adapt their plans in response to local opinions and concerns and to put trials on hold until appropriate community authorization has been achieved.

## Planning for Success

Many new products face the notorious “valley of death” when moving from basic research to development, but for conceptually novel or unprecedented products, the challenges of crossing that valley may seem especially daunting. As for any adventure, it will be important to be as prepared as possible to begin the journey and as resourceful as possible during it. Moving from the lab to the field with a novel vector control tool requires a multidisciplinary effort and capability for addressing not only technical but also complex regulatory and ethical, social, or cultural issues. Researchers can build on practices and policies in place from other areas but often will find themselves breaking new ground. Transparency, creativity, and consensus building will be critical to success.

**Key Learning Points**

1. Translation of conceptually novel vector control technologies to field application will be a multidisciplinary effort that requires capability and resources for addressing not only scientific challenges but also complex regulatory and ethical/social/cultural issues.
2. Where a truly novel product is proposed, there may be little or no regulatory precedent, which will necessitate a mechanism for capacity strengthening of the biosafety and regulatory infrastructure.
3. Novel products will be associated with uncertainties, which require risk assessment and risk management that considers not only potential biological and ecological effects but also broader social and economic concerns.
4. Testing of vector control tools that function on an area-wide level will require interaction with individuals who live in the vicinity but do not meet the defined criteria for research subjects; community engagement processes allow researchers to meet their obligations to this larger group of stakeholders.
5. Acceptability of the research and the intended product is key to success; ensuring local ownership of the product development and stakeholder engagement process, with appropriate training and technology transfer, is fundamental to product acceptance.

## References

[1] Wilson AL, Boelaert M, Kleinschmidt I, Pinder M, Scott TW, et al Evidence-based vector control? Improving the quality of vector control trials. Trends Parasitol. 2015;31(8):380–390. Epub 2015/05/23. 10.1016/j.pt.2015.04.015 . 25999026

[2] World Health Organization. The Guidance Framework for testing genetically modified mosquitoes 2014 http://www.who.int/tdr/publications/year/2014/guide-fmrk-gm-mosquit/en/.

[3] Brown DM, Alphey LS, McKemey A, Beech C, James AA. Criteria for identifying and evaluating candidate sites for open-field trials of genetically engineered mosquitoes. Vector Borne Zoonotic Dis. 2014;14(4):291–299. Epub 2014/04/03. 10.1089/vbz.2013.1364 24689963 PMC3993056

[4] American Committee of Medical Entomology. Arthropod Containment Levels. Vector-Borne and Zoonotic Diseases. 2004;3(2):75–90. 10.1089/153036603322163439

[5] Benedict M, D'Abbs P, Dobson S, Gottlieb M, Harrington L, et al Guidance for contained field trials of vector mosquitoes engineered to contain a gene drive system: recommendations of a scientific working group. Vector Borne Zoonotic Dis. 2008;8(2):127–166. Epub 2008/05/03. 10.1089/vbz.2007.0273 . 18452399

[6] Ramsey JM, Bond JG, Macotela ME, Facchinelli L, Valerio L, et al A regulatory structure for working with genetically modified mosquitoes: lessons from Mexico. PLoS Negl Trop Dis. 2014;8(3):e2623 Epub 2014/03/15. 10.1371/journal.pntd.0002623 24626164 PMC3952825

[7] European Food Safety Authority. Guidance on the environmental risk assessment of genetically modified animals. 2013 http://www.efsa.europa.eu/en/efsajournal/pub/3200

[8] Convention on Biological Diversity Bio-safety Clearing-House. Guidance on risk assessment of living modified organisms. 2014 https://bch.cbd.int/protocol/guidance_risk_assessment/.

[9] Convention on Biological Diversity Bio-safety Clearing-House. Risk assessment of Aedes aegypti strain OX513. 2014 https://bch.cbd.int/database/record.shtml?documentid=105831.

[10] Eliminate Dengue Program. Risk analysis on the Australian release of Aedes aegypti (L.) (Diptera: Culicidae) containing Wolbachia. 2010.http://www.eliminatedengue.com/library/publication/document/csiro_report_australia_2010.pdf

[11] Department of Health and Human Services. Establishment by the National Institutes of Health of Categorical Exclusions Under the National Environmental Policy Act 2000. http://www.gpo.gov/fdsys/pkg/FR-2000-01-19/html/00-1128.htm.

[12] Kolopack PA, Parsons JA, Lavery JV. What makes community engagement effective?: Lessons from the Eliminate Dengue Program in Queensland Australia. PLoS Negl Trop Dis. 2015;9(4):e0003713 Epub 2015/04/16. 10.1371/journal.pntd.0003713 25875485 PMC4395388

[13] Kahan D. Why we are poles apart on climate change. Nature. 2012;488(7411):255 Epub 2012/08/17. 10.1038/488255a . 22895298

